# Supplementary material for: Single [0001]-oriented zinc metal anode enables sustainable zinc batteries
Source: Nat Commun. 2024 Mar 28;15:2735. doi: 10.1038/s41467-024-47101-1 (PMC10978850; doi:10.1038/s41467-024-47101-1)
Supplement: Supplementary file 1 — Supplementary information [file 41467_2024_47101_MOESM1_ESM.pdf]

Supporting Information for

Single [0001]-oriented zinc metal anode enables sustainable zinc batteries

Xiaotan Zhang[1#], Jiangxu Li[2,3#], Yanfen Liu[1], Bingan Lu[4], Shuquan Liang[1]* & Jiang Zhou[1]*

[1]School of Materials Science and Engineering, Central South University, Changsha, Hunan 410083, P. R. China

[2]Department of Applied Chemistry, University of Science and Technology of China, Hefei, Anhui 230026, P. R. China

[3]Shenyang National Laboratory for Materials Science, Institute of Metal Research, Chinese Academy of Sciences, Shenyang 110016, P. R. China

[4]School of Physics and Electronics, Hunan University, Changsha, Hunan 410082, P. R. China

[#]These authors contributed equally: Xiaotan Zhang, Jiangxu Li.

*Corresponding authors: lsq@csu.edu.cn; zhou_jiang@csu.edu.cn

Supplementary Figures

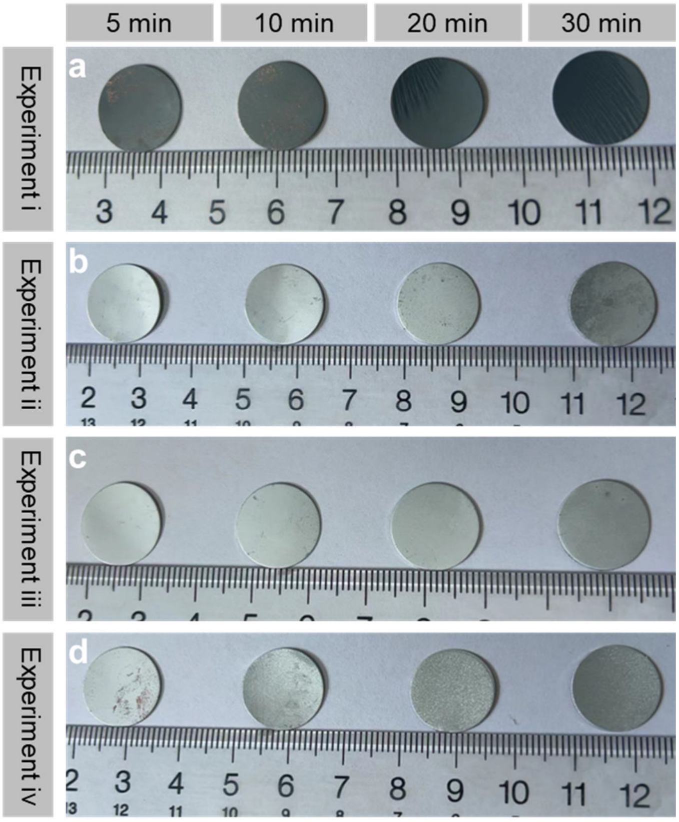

Supplementary Fig. 1. Macroscopic morphology photographs of electrodeposited Zn metal electrodes under the corresponding electrodeposition parameters of Supplementary Table 1. Experiment (a) i, (b) ii, (c) iii and (d) iv.

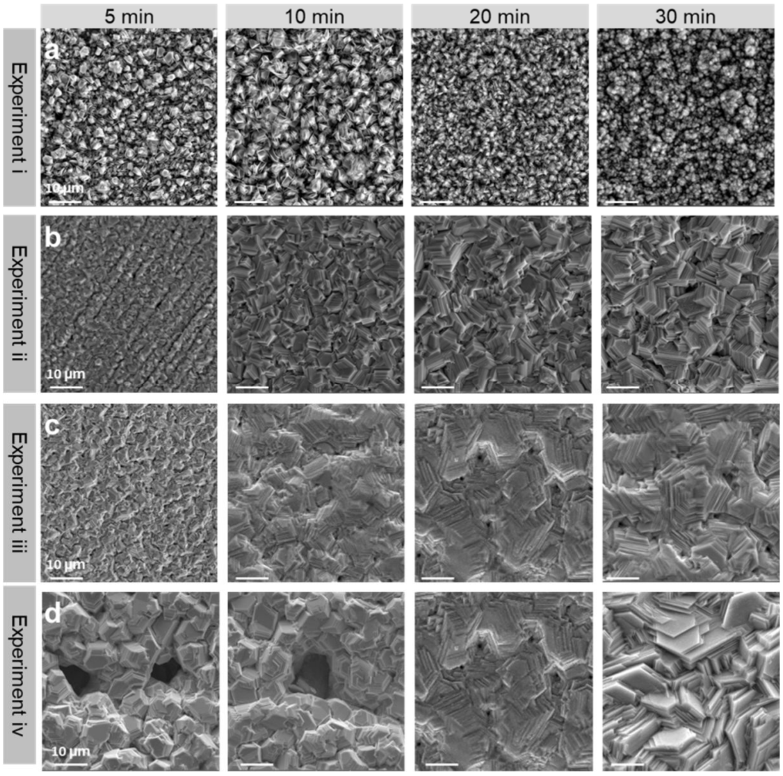

Supplementary Fig. 2. SEM images of electrodeposited Zn metal electrodes under the corresponding electrodeposition parameters of Supplementary Table 1. Experiment (a) i, (b) ii, (c) iii and (d) iv.

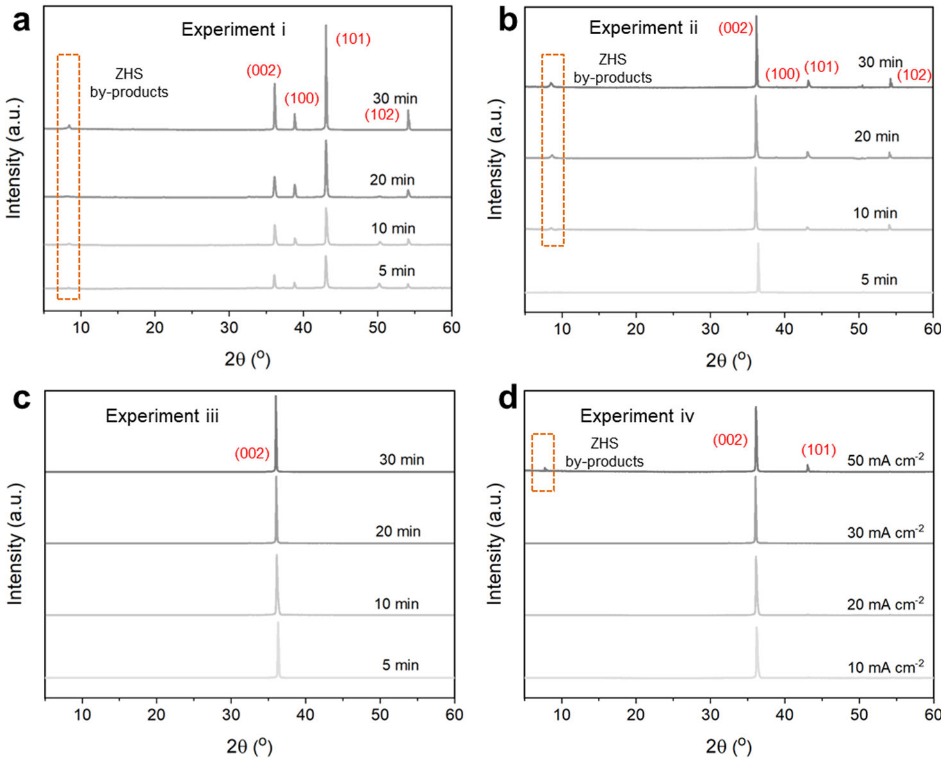

Supplementary Fig. 3. XRD patterns of electrodeposited Zn metal electrodes under the corresponding electrodeposition parameters of Supplementary Table 1. Experiment (a) i, (b) ii, (c) iii and (d) iv.

To comprehensively investigate the impact of electroplating parameters on the metal texture of electrodeposited Zn electrodes, a series of experiments were conducted (**Supplementary Table 1**), outlined as follows:

i) Under 30 mA cm^{-2} current density and vigorous stirring, electrodeposited Zn metal electrodes in 100 g L^{-1} $ZnSO_4$ solution formed $Zn_4SO_4(OH)_6{\cdot}5H_2O$ (ZHS) by-products due to severe side reactions, resulting in a disordered Zn texture (**Supplementary Fig. 1a, Supplementary Fig. 2a, and Supplementary Fig. 3a**).

ii) Under 30 mA cm^{-2} current density and vigorous stirring, electrodeposited Zn metal electrodes in 100 g L^{-1} $ZnSO_4$ solution with pH = 2 initially maintained a single crystalline orientation. However, prolonged deposition led to a decrease in pH value, triggering enhanced side reactions and reducing deposition efficiency, resulting in a disordered texture and uneven surface (**Supplementary Fig. 1b, Supplementary Fig. 2b, and Supplementary Fig. 3b**).

iii) Under 30 mA cm^{-2} current density and vigorous stirring, electrodeposited Zn metal electrodes

in 100 g L^{-1} $ZnSO_4$ and 20 g L^{-1} H_3BO_3 solution with pH = 2 exhibited a single Zn(0002) texture without by-products (**Supplementary Fig. 1c, Supplementary Fig. 2c, and Supplementary Fig. 3c**). This was attributed to H_3BO_3 incorporation and vigorous stirring, facilitating maximum exposure of the Zn(0002) crystal plane with the lowest surface energy.

iv) Regulating the current density within Experiment iii parameters revealed intriguing results. Lower currents (10 and 20 mA cm^{-2}) facilitated fabrication of Zn metal electrodes with a single (0002) texture, accompanied with reduced deposition efficiency and surface unevenness (**Supplementary Fig. 1d and Supplementary Fig. 2d**). Conversely, higher current density (50 mA cm^{-2}) exacerbated side reactions and uncontrolled Zn growth, resulting in a disordered Zn texture (**Supplementary Fig. 2d and Supplementary Fig. 3d**). Thus, a current density of 30 mA cm^{-2} appears optimal.

In summary, stirring, H_3BO_3 additives, pH values, and appropriate current density are crucial in establishing an optimal electroplating environment for fabricating a single (0002)-textured Zn metal electrode.

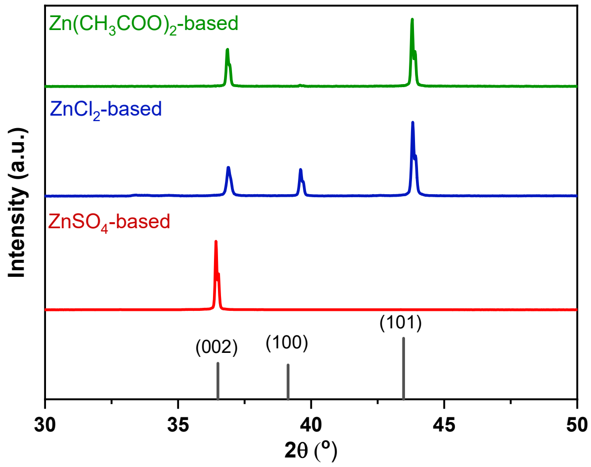

Supplementary Fig. 4 | XRD patterns of the electrodeposited Zn metal electrodes in $ZnSO_4$-, $ZnCl_2$-, and $Zn(CH_3COO)_2$-based systems.

The XRD pattern of the Zn metal electrode electrodeposited in a $ZnSO_4$-based solution only presented a single (002) peak, while Zn metal electrodes obtained from $ZnCl_2$- and $Zn(CH_3COO)_2$-based solutions exhibited non-single crystal diffraction peaks. Notably, the Zn electrode deposited in the $Zn(CH_3COO)_2$-based solution only displayed a (101) peak, in addition to the (002) peak. This observation suggests a potential avenue for fabricating IMS-Zn(0002) metal electrodes ($RTC_{(002)} = 93$). Furthermore, when compared to the Zn electrode prepared from a $ZnSO_4$ electrolyte, the XRD peaks of the Zn electrodes electrodeposited from $Zn(CH_3COO)_2$ and $ZnCl_2$-based electrolytes exhibited a rightward shift of 0.36°. According to the Bragg equation ($2d \sin\theta = \lambda$), an increase in θ results in a reduced interplanar spacing (d), indicating lattice contraction. Consequently, the observed rightward peak shift of Zn metal electrodes deposited from $Zn(CH_3COO)_2$ and $ZnCl_2$-based electrolytes further demonstrate their disordered crystalline orientation.

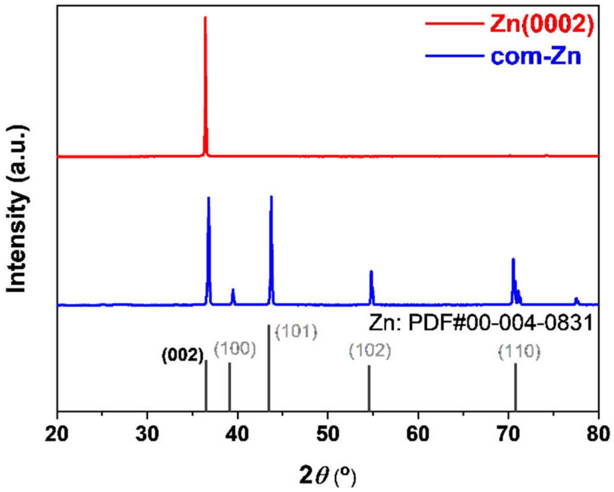

Supplementary Fig. 5 | XRD patterns of the com-Zn and as-deposited Zn(0002) metals. The XRD pattern of the com-Zn revealed disordered metal textures characteristic of Zn crystal (PDF#00-004-0831), whereas the XRD pattern of Zn(0002) displayed a prominent, single peak corresponding to (002) located at 36°.

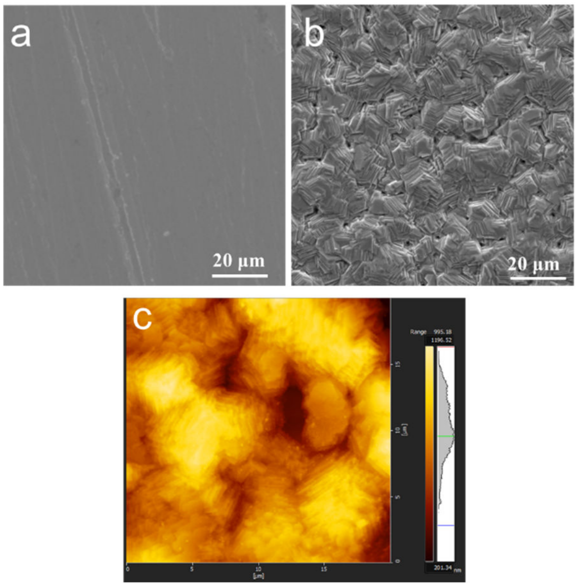

Supplementary Fig. 6 | Surface morphology of com-Zn and Zn(0002) metal electrodes. SEM images of (a) the com-Zn and (b) the as-deposited Zn(0002) metals. (c) Atomic force microscope (AFM) image of the as-deposited Zn(0002) metal.

The polished surface of com-Zn electrode showed a minimal number of scratches, while the electrodeposited Zn(0002) electrodes displayed a layered structure stacked of hexagonal sheets.

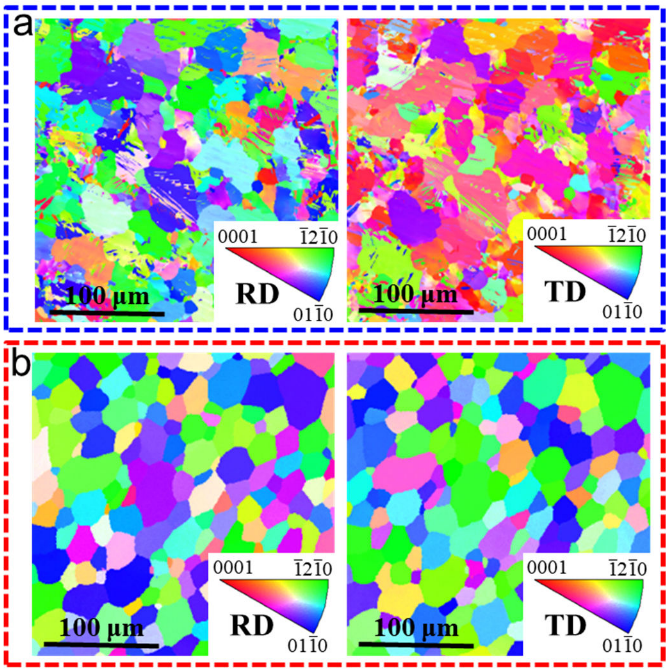

Supplementary Fig. 7 | EBSD characterization of Zn electrodes. Orientation maps of (a) com-Zn and (b) Zn(0002) metals along rolling direction (RD) and transection direction (TD). Compared to com-Zn metal electrodes, the Zn(0002) electrodes displayed a highly organized crystalline orientation along RD, TD, ang ND.

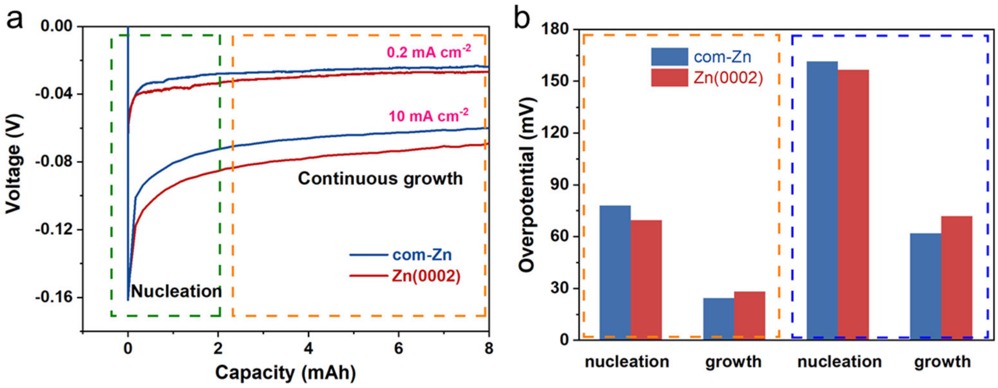

Supplementary Fig. 8 | The nucleation and growth overpotential of Zn electrodes. (a) Experimental voltage profiles of Zn deposition on com-Zn and Zn(0002) electrodes at low or high current density for a total capacity of 8 mAh. (b) The corresponding nucleation overpotential and plateau overpotential in (a).

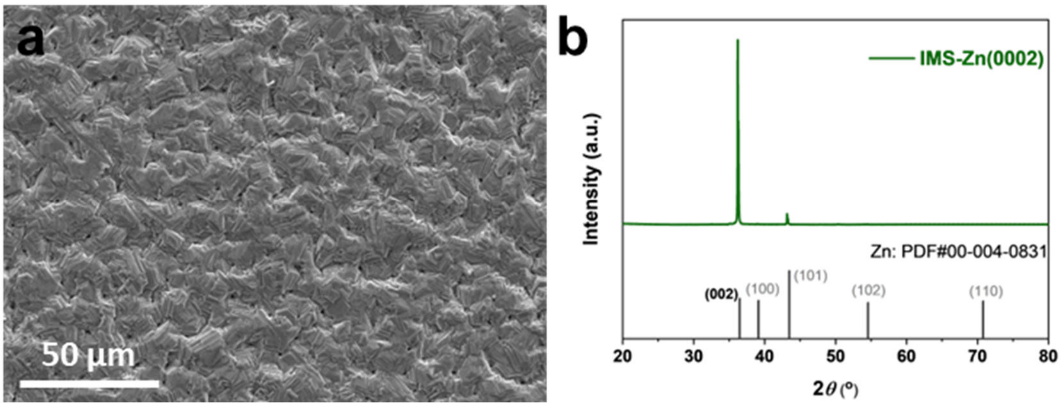

Supplementary Fig. 9 | The electrodeposited IMS-Zn(0002) metal electrodes. (a) SEM image and (b) XRD pattern of the electrodeposited IMS-Zn(0002) metal electrode.

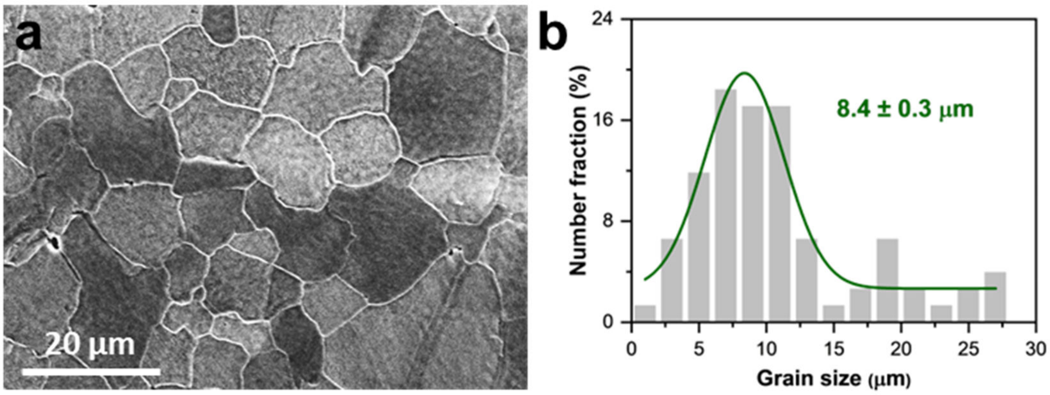

Supplementary Fig. 10 | The SEM image and the corresponding grain size of the IMS-Zn(0002) metal electrodes. (a) SEM image of the polished IMS-Zn(0002) metal electrode. (b) Grain size distributions from statistical SEM measurement for the IMS-Zn(0002) metal electrode.

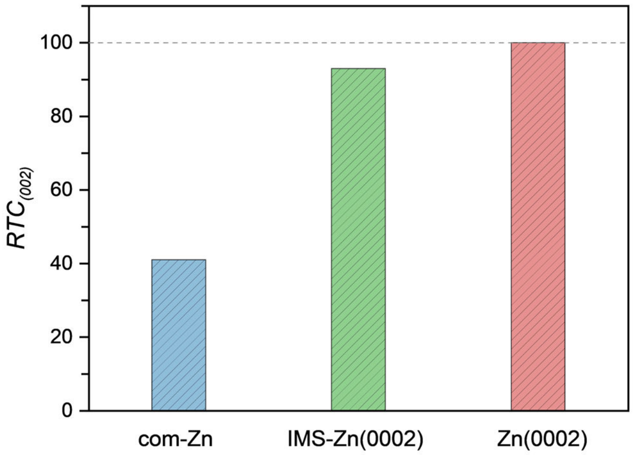

Supplementary Fig. 11 | The ratio of Zn(0002) texture of Zn electrodes. The calculated $RTC_{(002)}$ value of com-Zn, IMS-Zn(0002) and Zn(0002) metal electrodes.

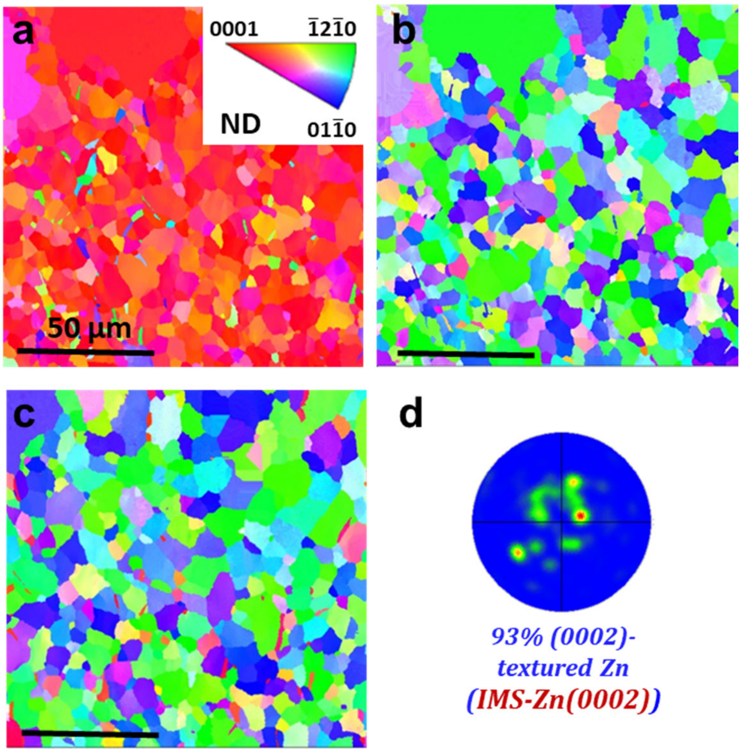

Supplementary Fig. 12 | The EBSD characterization of IMS-Zn(0002) metal electrodes. Orientation maps of IMS-Zn(0002) metals along (a) normal direction (ND), (b) rolling direction (RD) and (c) transection direction (TD). (d) The corresponding (0002) pole figure.

There are a small amount of grains deviated from [0001] crystalline orientation in IMS-Zn(0002) metal electrodes.

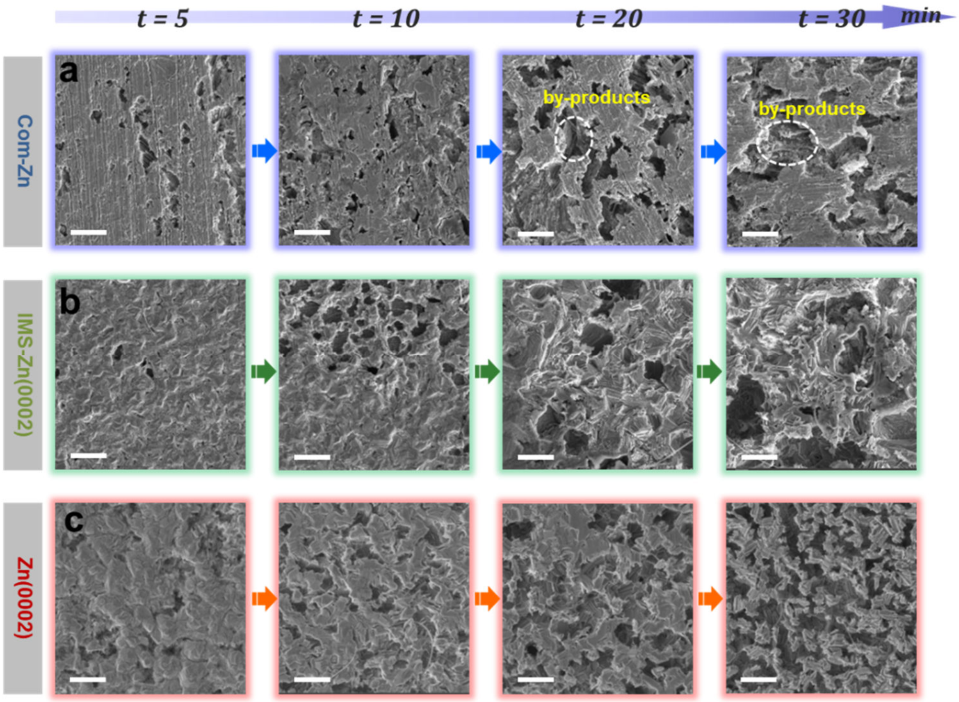

Supplementary Fig. 13 | The morphology evolution of Zn metal electrodes during stripping. SEM images of (a) com-Zn, (b) IMS-Zn(0002) and (c) Zn(0002) electrodes after stripping from 5 to 30 min (scale bar, 20 μm). Current density, $J = 4$ mA cm^{-2}.

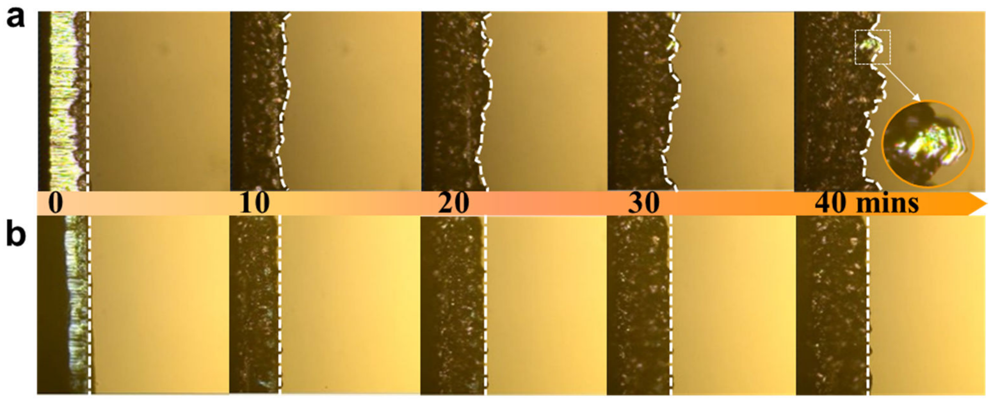

Supplementary Fig. 14 | The deposition morphology of Zn metal electrodes during plating. In situ optical microscopy investigation of Zn plating behaviors on the surface of (a) com-Zn and (b) as-deposited Zn(0002) metals.

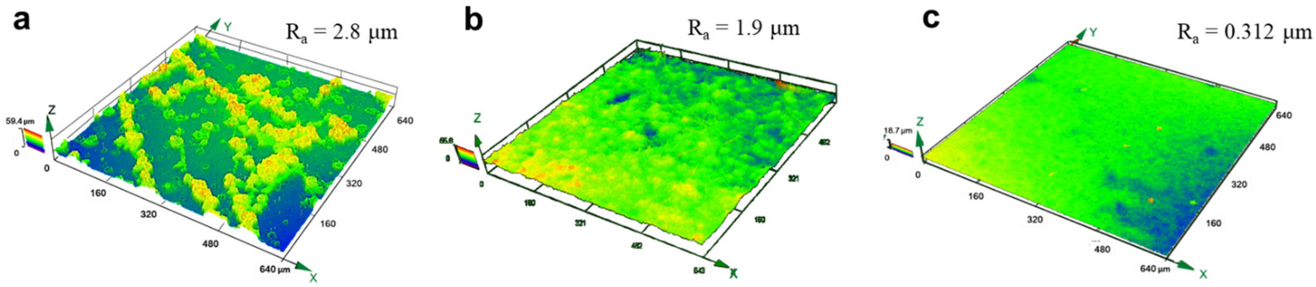

Supplementary Fig. 15 | Surface roughness of Zn metal electrodes after plating. LSM images of (a) com-Zn, (b) IMS-Zn(0002) and (c) Zn(0002) electrodes after plating 1 h.

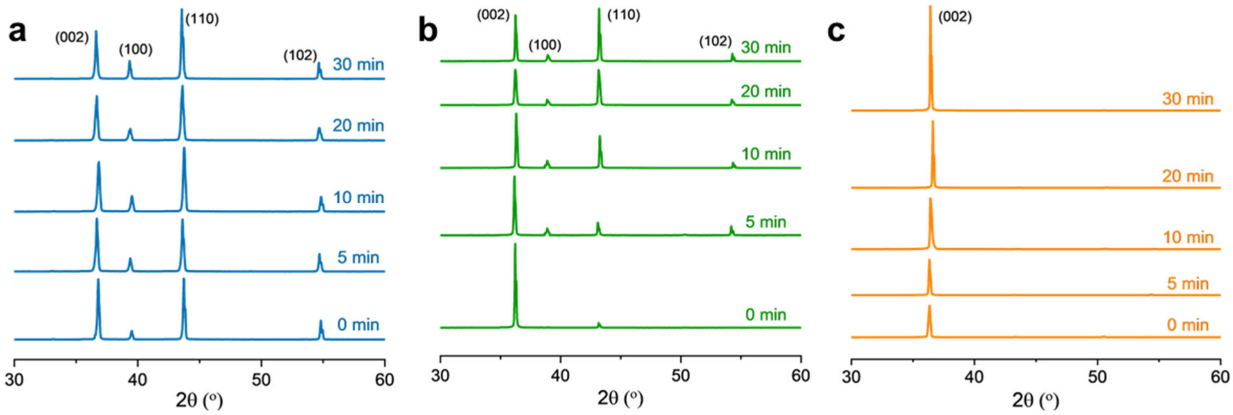

Supplementary Fig. 16 | Structure evolution of Zn metal electrodes during plating process. XRD patterns of Zn electrodes after plating 0, 5, 10, 20, 30 min, respectively. (a) com-Zn, (b) IMS-Zn(0002) and (c) Zn(0002).

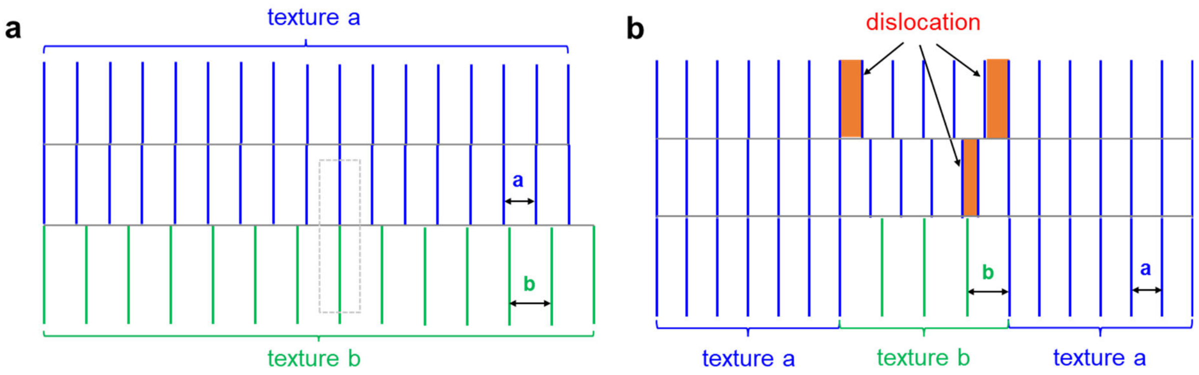

Supplementary Fig. 17 | Mechanism diagram of substrate crystals with/without a single texture. Schematic diagram of lattice match between substrate crystal and overgrowth crystal. Epitaxial substrate with (a) single crystalline orientation and (b) non-single crystalline orientation.

As illustrated in Supplementary Fig. 17a, if there is a tolerable lattice mismatch between the substrate crystal and the overgrowth crystal, and the substrate crystal possesses a single crystalline orientation, the subsequently deposited crystals exhibit the anticipated crystalline orientation. However, in our study, we emphasize that the prepared Zn(0002) metal anodes only possesses a singular crystalline orientation to achieve ultra-sustainable homoepitaxial growth. The presence of even a small amount of other Zn textures in the Zn(0002) metal anode will result in the formation of dislocations (as shown in the orange region in Supplementary Fig. 17b), leading to lattice distortion. Once the thickness of the deposition layer surpasses the critical value for epitaxial growth, the subsequently deposited crystals display a disordered crystalline orientation, indicating the failure of the epitaxial mechanism.

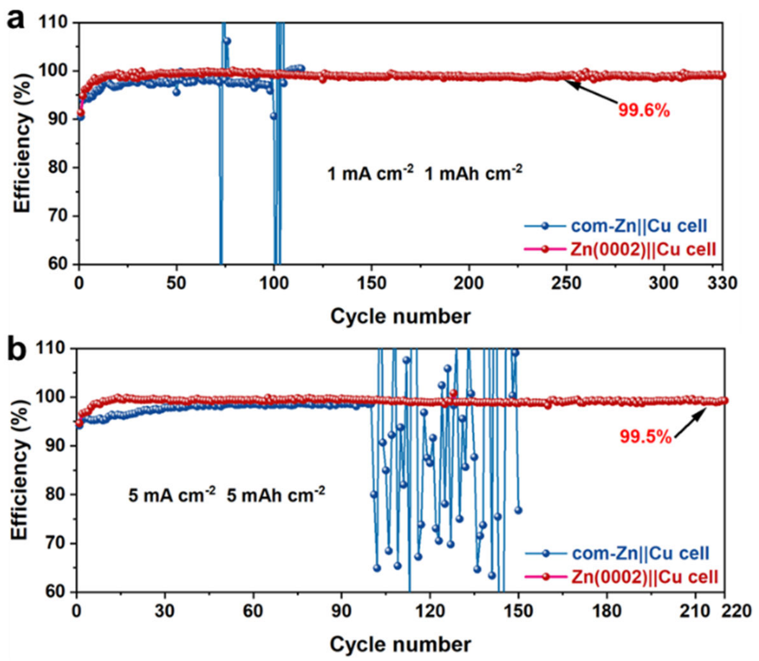

Supplementary Fig. 18 | Coulombic efficiencies of Zn metal electrodes. Coulombic efficiencies of Zn plating/stripping process in com-Zn||Cu and Zn(0002)||Cu cells at (a) 1 mA cm^{-2}/1 mAh cm^{-2} and (b) 5 mA cm^{-2}/5 mAh cm^{-2}.

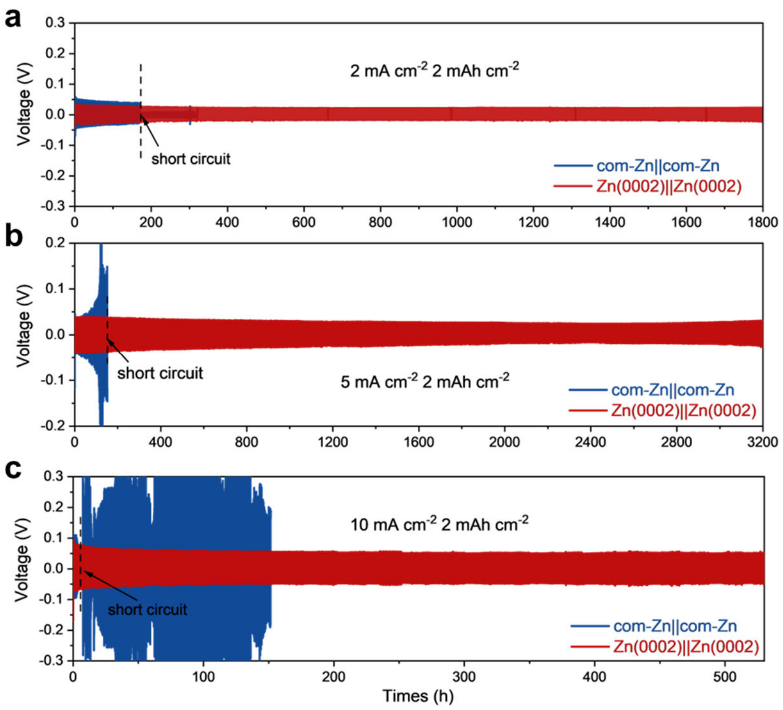

Supplementary Fig. 19 | Long-term galvanostatic cycling of Zn||Zn symmetric cells using com-Zn and Zn(0002) electrodes with a current density of (a) 2 mA cm^{-2}, (b) 5 mA cm^{-2} and (c) 10 mA cm^{-2}, respectively.

Under a fixed capacity of 2 mAh cm^{-2}, the Zn(0002) symmetric cells can stably cycle for 1800 h (2 mA cm^{-2}), 3200 h (5 mA cm^{-2}) and 550 h (10 mA cm^{-2}), respectively. In comparison to the com-Zn metal electrode, the Zn(0002) metal electrode exhibits exceptional corrosion resistance performance.

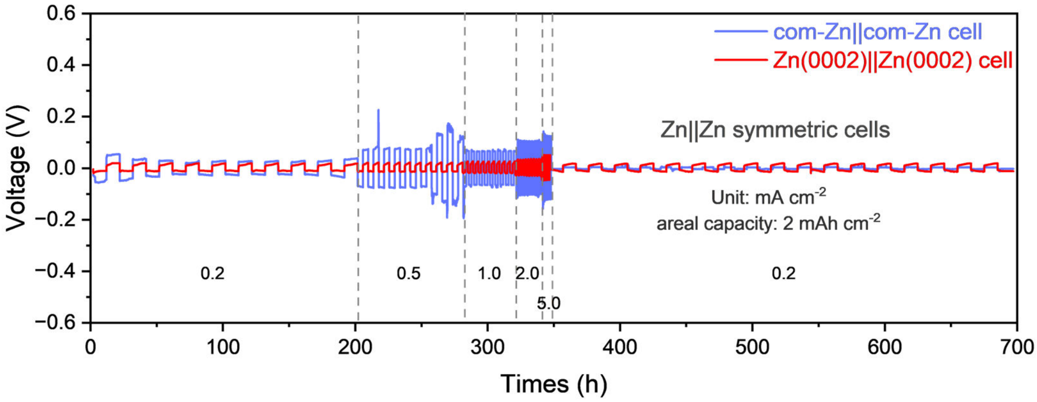

Supplementary Fig. 20 | Rate performances of Zn symmetric cells. Rate performances for the com-Zn||com-Zn and Zn(0002)||Zn(0002) cells at various current densities and a capacity of 2 mAh cm^{-2}.

Zn(0002)||Zn(0002) cells show superior rate performance in comparison with bare Zn||bare Zn cells. When enhancing the current densities from 0.2 to 5 mA cm^{-2} and then decreasing to 0.2 mA cm^{-2}, Zn(0002)||Zn(0002) cells still present stable voltage profiles with low overpotential of 37, 41, 46, 62, 99 and 36 mV, at 0.2, 0.5, 1, 2, 5, 0.2 mA cm^{-2}, respectively.

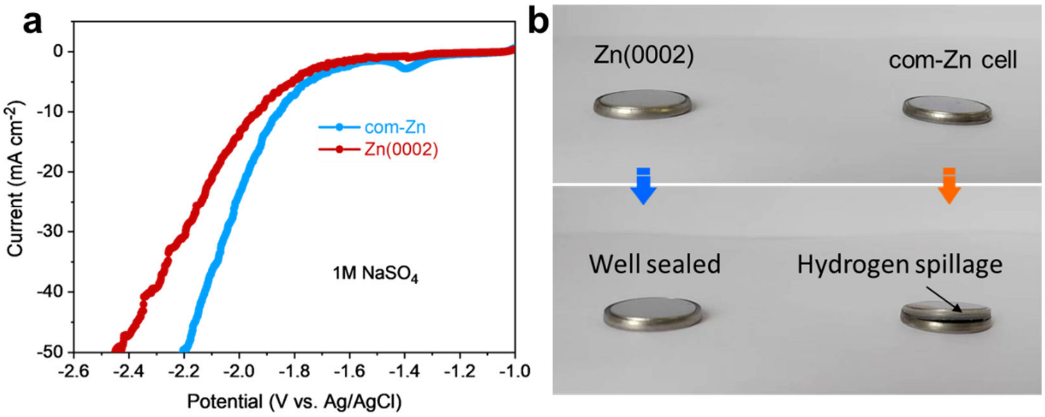

Supplementary Fig. 21 | Hydrogen evolution rates of Zn metal electrodes. (a) LSV curves of the com-Zn and Zn(0002) electrodes in 1 M Na_2SO_4 solution at a scan rate of 5 mV s^{-1}. (b) Optical images of Zn||Zn symmetric cells after plating/stripping at 2 mA cm^{-2}/5 mAh cm^{-2} for 200 cycles.

The potential of HER was tested in 1 M Na_2SO_4 aqueous solutions in order to avoid the interference of Zn reduction. Furthermore, owing to the constant generation of H_2 by water consumption, the cell based on com-Zn expands and detaches, causing electrolyte leakage and early battery failure.

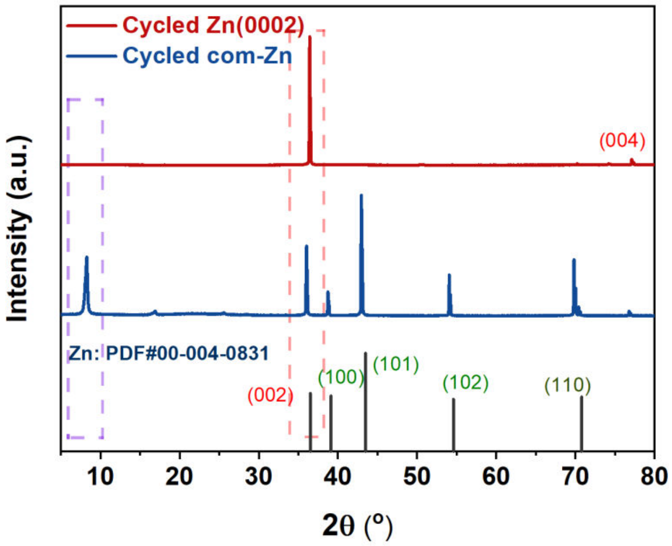

Supplementary Fig. 22 | Corrosion by-products on the surface of cycled Zn electrodes. XRD patterns of com-Zn and Zn(0002) electrodes in Zn||Zn cells after cycling 100 h.

After cycling 100 h of Zn symmetric cells, the XRD pattern of com-Zn electrode showed that a new peak, corresponding to the (002) plane of ZHS (PDF#00-039-0688), disappeared for Zn(0002), demonstrating its superior corrosion resistance.

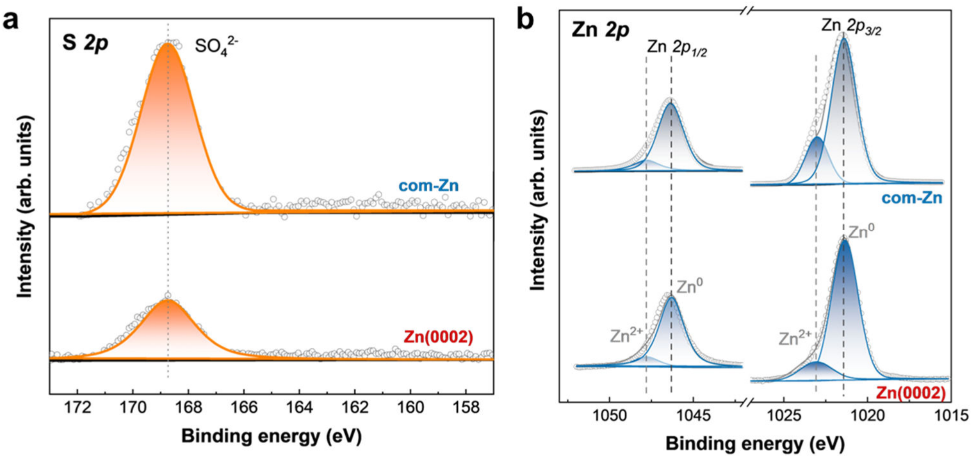

Supplementary Fig. 23 | (a) S *2p* and (b) Zn *2p* XPS spectra of Zn electrodes after cycling.

The application of further XPS analysis unveils the surface chemical composition. The S *2p* XPS spectra indicate distinct SO_4^{2-} peaks at 168.7 eV on the surface of Zn anodes after cycling, with significantly higher intensity observed on bare Zn. Meanwhile, the Zn $2p_{3/2}$ XPS spectra can be deconvoluted into two peaks at 1023.3 eV and 1021.8 eV, corresponding to Zn^{2+} and Zn^0, respectively. Notably, the proportion of Zn^{2+} for com-Zn (20.3%) exceeds that in the Zn(0002) alloy (11.3%). These findings suggest the Zn(0002) metal electrode shows superior corrosion resistance than com-Zn electrode.

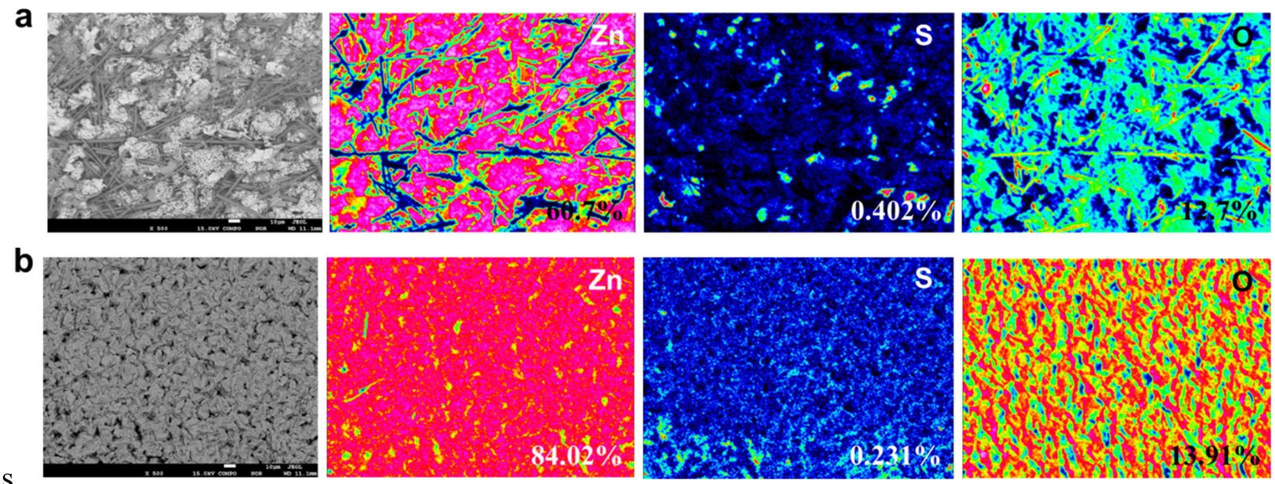

s

Supplementary Fig. 24 | EPMA-WDS images of (a) com-Zn and (b) Zn(0002) electrodes in Zn||Zn cells after cycling 100 h.

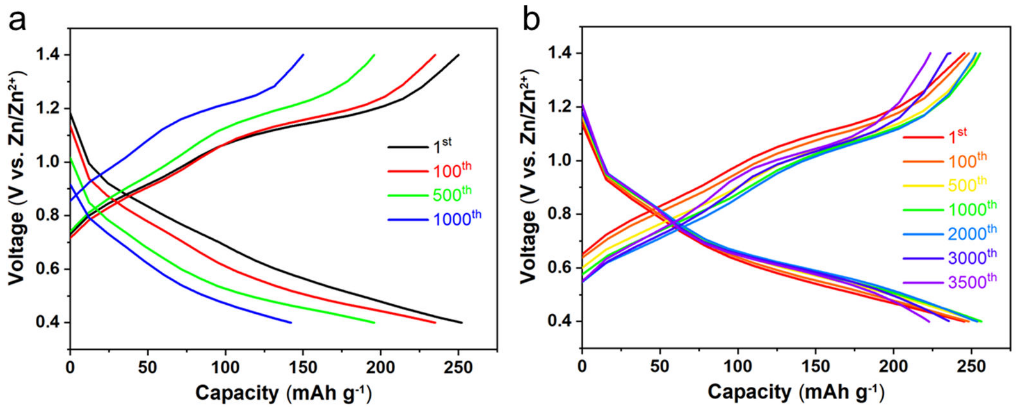

Supplementary Fig. 25 | Charge-discharge curves of the (a) com-Zn||NVO and (b) Zn(0002)||NVO cells at 5 A g^{-1}.

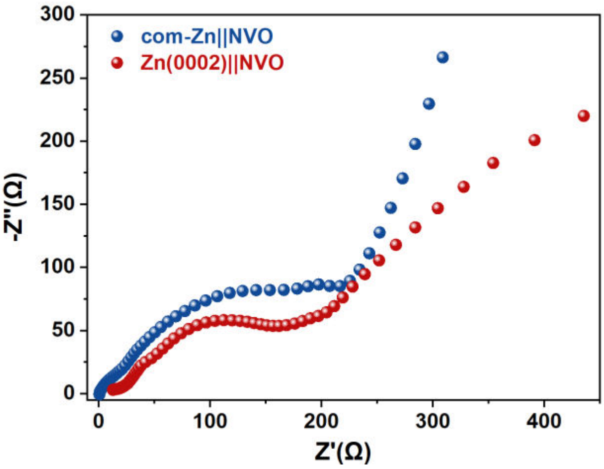

Supplementary Fig. 26 | Electrochemical impedance spectra curves of the com-Zn||NVO and the Zn(0002)||NVO cells after 1000 cycles.

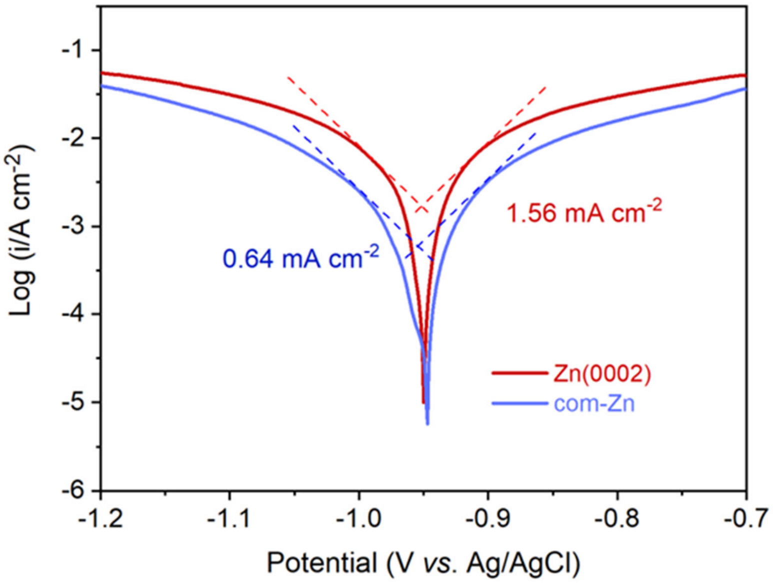

Supplementary Fig. 27 | Measurement of exchange current density of Zn electrode. Linear polarization curves of com-Zn and Zn(0002) electrodes by a 3-electrode configuration.

According to the Butler-Volmer equation, the expression of the charge-transfer resistance (R_{ct}) changes info:

$$R_{ct} = \frac{RT}{nFi_0} \tag{1}$$

Where R is gas constant, T is temperature, F is Faraday's constant, n is number of electrons involved, and i_0 is exchange-current density. From this equation the R_{ct} is related to i_0, the larger the exchange current density, the smaller the interface charge transfer resistance. We conducted a half-cell experiment on the Zn electrode with a 3-electrode configuration to analyze the i_0 by Tafel curves. The i_0 depends critically on the nature of the electrode. The Zn(0002) exhibits the high exchange current density of 1.56 mA cm^{-2}, whereas the com-Zn presents the exchange current density of 0.64 mA cm^{-2}, indicating the fast deposition kinetic of the Zn(0002).

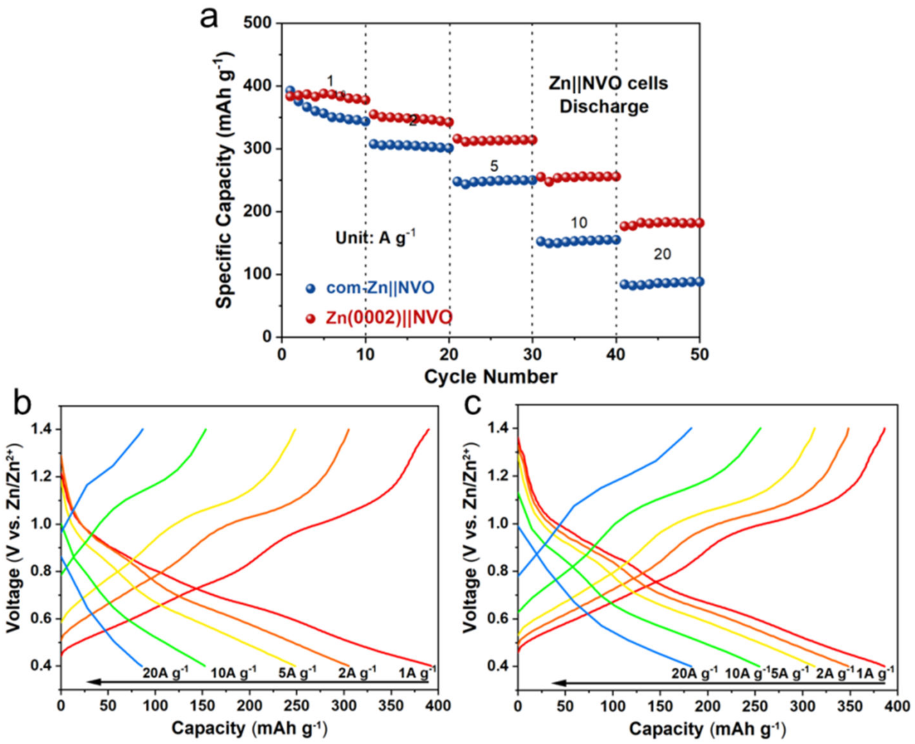

Supplementary Fig. 28 | (a) Rate capability of com-Zn||NVO and Zn(0002)||NVO cells at varied current densities from 1 to 20 A g^{-1}. The corresponding charge-discharge curves of the Zn||NVO cells with (b) com-Zn and (c) Zn(0002) anodes.

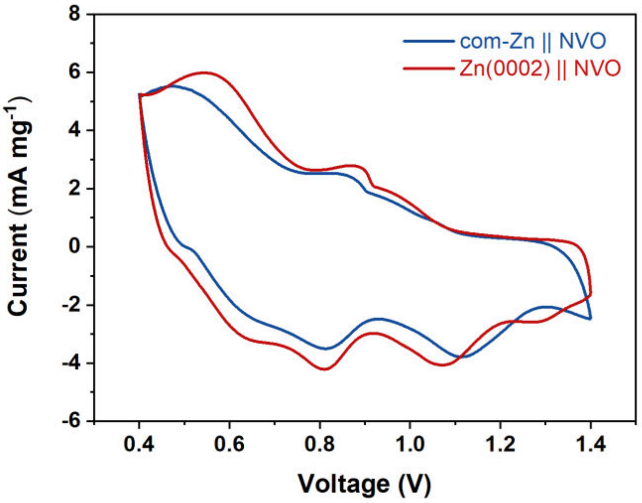

Supplementary Fig. 29 | CV curves of com-Zn||NVO and Zn(0002)||NVO cells at 1 mV s^{-1}.

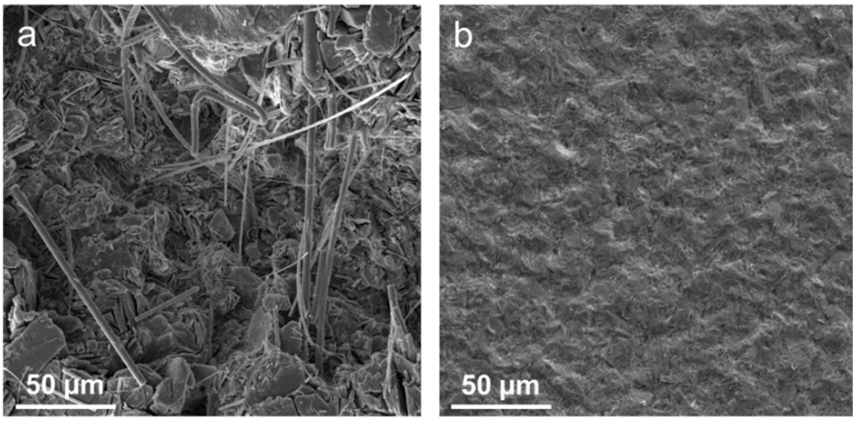

Supplementary Fig. 30 | Top-view SEM images of (a) com-Zn and (b) Zn(0002) metal anodes in Zn||NVO cells after 1000 cycles.

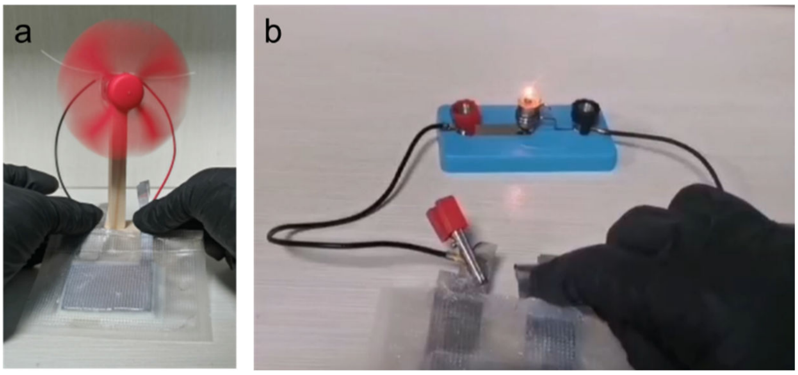

Supplementary Fig. 31 | The multiple-layer Zn(0002)||NVO pouch cell with fully charged state (a) turning a fan (Supplementary Movie 1) and (b) illuminating a bulb (Supplementary Movie 2), respectively.

Supplementary Table 1. The electrodeposition parameters of Zn metal electrodes

Experiment number	i	ii	iii	iv
Electrodeposition parameters	100 g L^{-1} $ZnSO_4·6H_2O$, *J* = 30 mA cm^{-2}	100 g L^{-1} $ZnSO_4·6H_2O$, **pH =2**, *J* = 30 mA cm^{-2}	100 g L^{-1} $ZnSO_4·6H_2O$, pH =2, **20 g L^{-1} H_3BO_3**, *J* = 30 mA cm^{-2}	100 g L^{-1} $ZnSO_4·6H_2O$, pH =2, 20 g L^{-1} H_3BO_3, ***J* = 10~50 mA cm^{-2}**
