# Supplementary material for: Single [0001]-oriented zinc metal anode enables sustainable zinc batteries
Source: Nat Commun. 2024 Mar 28;15:2735. doi: 10.1038/s41467-024-47101-1 (PMC10978850; doi:10.1038/s41467-024-47101-1)
Supplement: Supplementary file 3 — Description of Additonal Supplementary Files [file 41467_2024_47101_MOESM3_ESM.pdf]

Description of Additional Supplementary Files

Supplementary Movie 1

Description: The assembled multi-layer pouch cells to power a fan.

Supplementary Movie 2

Description: The assembled multi-layer pouch cells to light up a bulb.
